# Repair of 3-methyladenine and abasic sites by base excision repair mediates glioblastoma resistance to temozolomide

**DOI:** 10.3389/fonc.2012.00176

**Published:** 2012-11-30

**Authors:** Michael S. Bobola, Douglas D. Kolstoe, A. Blank, Marc C. Chamberlain, John R. Silber

**Affiliations:** ^1^Department of Neurological Surgery, University of Washington Medical CenterSeattle, WA, USA; ^2^Department of Neurology, University of Washington Medical CenterSeattle, WA, USA

**Keywords:** alkyladenine-DNA glycosylase, Ape1, apurinic endonuclease, DNA repair, treatment outcome, predictive marker

## Abstract

Alkylating agents have long played a central role in the adjuvant therapy of glioblastoma (GBM). More recently, inclusion of temozolomide (TMZ), an orally administered methylating agent with low systemic toxicity, during and after radiotherapy has markedly improved survival. Extensive *in vitro* and *in vivo* evidence has shown that TMZ-induced O^6^-methylguanine (O^6^-meG) mediates GBM cell killing. Moreover, low or absent expression of O^6^-methylguanine-DNA methyltransferase (MGMT), the sole human repair protein that removes O^6^-meG from DNA, is frequently associated with longer survival in GBMs treated with TMZ, promoting interest in developing inhibitors of MGMT to counter resistance. However, the clinical efficacy of TMZ is unlikely to be due solely to O^6^-meG, as the agent produces approximately a dozen additional DNA adducts, including cytotoxic N3-methyladenine (3-meA) and abasic sites. Repair of 3-meA and abasic sites, both of which are produced in greater abundance than O^6^-meG, is mediated by the base excision repair (BER) pathway, and occurs independently of removal of O^6^-meG. These observations indicate that BER activities are also potential targets for strategies to potentiate TMZ cytotoxicity. Here we review the evidence that 3-meA and abasic sites mediate killing of GBM cells. We also present *in vitro* and *in vivo* evidence that alkyladenine-DNA glycosylase, the sole repair activity that excises 3-meA from DNA, and Ape1, the major human abasic site endonuclease, mediate TMZ resistance in GBMs and represent potential anti-resistance targets.

## INTRODUCTION

Methylating and chloroethylating agents have long been used in the adjuvant therapy of glioblastoma (GBM) and other malignant gliomas (Chamberlain, 2011). Inclusion of the methylator temozolomide (TMZ) during radiotherapy (RT) and continued administration of TMZ as a single agent afterward produces significant improvement in survival, marking a milestone in neuro-oncology (Stupp et al., 2009). Better outcome with concurrent TMZ-RT is associated with methylation of CpG dinucleotides in the promoter of the gene for O^6^-methylguanine-DNA methyltransferase (MGMT), indicative of silencing of expression of the sole human activity that removes TMZ-induced radiosensitizing and cytotoxic O^6^-methylguanine (O^6^-meG) adducts from DNA (Hegi et al., 2005; Stupp et al., 2009). This observation has raised expectations that *MGMT* promoter methylation status can be used to direct treatment of individual tumors (e.g., Weller et al., 2012). However, promoter methylation status does not accurately predict outcome in all GBM, indicating that additional intrinsic factors influence survival (Silber et al., 2012). Importantly, TMZ produces a host of potentially cytotoxic DNA adducts in addition to O^6^-meG that are not repaired by MGMT (Shrivastav et al., 2010; Fu et al., 2012). Here we summarize evidence that excision of N3-methyladenine (3-meA) by alkyladenine-DNA glycosylase (AAG), and repair of abasic sites by the apurinic/apyrimidinic endonuclease (Ap endo) activity of Ape1, also mediate GBM resistance to TMZ and other alkylators.

## TMZ PRODUCES A VARIETY OF CYTOTOXIC DNA BASE ADDUCTS

Temozolomide and other clinically relevant methylating agents (e.g., procarbazine, streptozotocin) are S_N_1-type alkylators that undergo spontaneous hydrolysis at physiological pH to form a methyldiazonium ion that reacts at nucleophilic ring nitrogens and extra-cyclic oxygens on purine and pyrimidines (Fu et al., 2012). In double-stranded DNA, reaction occurs predominantly at the N7 of guanine, N3 of adenine and O^6^ of guanine, accounting for approximately 70, 10, and 7% of base adducts, respectively (**Table 1**). The next two most frequent sites of adduction, N1 of adenine and N3 of cytosine (**Table 1**), are unusual in that they occur predominantly in single-stranded DNA such as would be found at DNA replication forks and at sites of gene transcription *in vivo* (Sedgwick et al., 2007). Methylation has also been detected at N1 and N3 of guanine, N7 of adenine, N3 of thymine, O^4^ of thymine, and O^2^ of cytosine, but these lesions comprise less than 5% of total base adducts in double-stranded DNA (Wyatt and Pittman, 2006). With the exception of the N3 position of adenine, clinically utilized, nitrosourea-derived chloroethylating agents (e.g., carmustine, lomustine) react at many of the same sites as TMZ, forming a number of potentially lethal monoadducts, exocyclic ethano adducts, and inter-strand cross-links, including the cytosine-guanine cross-link produced by O^6^-chloroethylguanine (Ludlum, 1997).

**Table 1:** Base adducts produced by S_N_1 methylating agents^a^.

	Percent	Biological effect	Disrupts	Repair
**Major adducts**
7-meG	70	Innocuous	–	AAG
3-meA	10	Cytotoxic	Polymerase contact	AAG
O^6^-meG	5–7	Cytotoxic	Base-pairing	MGMT
1-meA	2.8^b^	Cytotoxic	Polymerase contact	ABH2/ABH3
3-meC	2.3^b^	Cytotoxic	Polymerase contact	ABH2/ABH3
**Minor adducts**
7-meA	1.7	Innocuous	–	AAG
3-meG	0.8	Cytotoxic	Polymerase contact	AAG
O^4^-meT	0.4	Cytotoxic	Base-pairing	MGMT
1-meG	≪1^b^	Cytotoxic	Polymerase contact	ABH2/ABH3; AAG^c^
3-meT	≪1^b^	Cytotoxic	Polymerase contact	ABH2/ABH3
O^2^-meC	≪1	Cytotoxic	Base-pairing	?
O^2^-meT	≪1	Cytotoxic	Polymerase contact	?

a Compiled from Beranek (1990), Drabløs et al. (2004), Wyatt and Pittman (2006), Lee et al . (2009), and Shrivastav et al . (2010).

b In single-stranded DNA.

c In double-stranded DNA.

As shown in **Table 1**, most methyl base adducts are implicated in promoting lethality, and cytotoxicity is strongly associated with blocked or interrupted DNA replication (Fu et al., 2012). Cytotoxic methyl adducts occur at positions on bases that are contacted by DNA polymerases (e.g., N3 of adenine) or are involved in Watson–Crick base-pairing (e.g., O^6^ of guanine). Lesions such as 3-meA prevent the contacts with critical amino acid residues in replicative DNA polymerases that are obligatory for synthesis (Plosky et al., 2008; Sidorova, 2008; Myers et al., 2009). Blocked replication forks are unstable and produce potently cytotoxic double-strand breaks (DSBs) upon collapse. The commonly known lesion that disrupts base-pairing, O^6^-meG, does not block replication fork progression directly but allows incorporation of either cytosine or thymine, neither of which can correctly base-pair with the adduct (Roos and Kaina, 2012). The resulting mis-pair is recognized and the inserted base excised by the mismatch repair pathway, producing a long single-strand gap in newly synthesized DNA. Repair DNA synthesis to fill the gap produces another mis-pair, eliciting a futile cycle of excision and resynthesis, and the resulting single-strand gap produces a DSB during the next S-phase (Quiros et al., 2010). The single-stranded regions that result from replication blockage or the action of mismatch repair may also be responsible for the radiosensitizing effect of TMZ, since these sites are readily converted into DSBs by free radical-mediated strand cleavage (Bobola et al., 2010).

The most abundant TMZ base lesion, N7-methylguanine (7-meG), is innocuous, probably reflecting its position in the major groove of DNA that removes it from the path of DNA replication. However, methylation at the N7 position of guanine as well as at other sites in purines (e.g., N7 of adenine, N3 of guanine) can greatly accelerate the rate of hydrolysis of the glycosylic linkage that binds bases to deoxyribose (Loeb and Preston, 1986; Shrivastav et al., 2010). The resulting abasic sites are strong blocks to replication (Wilson and Barsky, 2001), and because of the preponderance of *N*-methyl adducts produced by methylating agents, are likely the most abundant potentially lethal lesion produced by TMZ.

## REPAIR OF METHYL BASE ADDUCTS IN DNA

A number of DNA repair pathways promote GBM resistance to TMZ-induced base adducts. The best characterized of these is MGMT, which functions solely to restore O^6^-meG to guanine by transferring the methyl group to an internal cysteine residue (Silber et al., 2012). However, the most abundant TMZ-induced adducts, 7-meG, 3-meA, and abasic sites are excised from DNA by the short patch base excision repair (BER) pathway, a carefully coordinated, multi-step process that replaces a single nucleotide containing a damaged base (Fu et al., 2012). BER is an evolutionarily conserved repair pathway that primarily functions against endogenously generated DNA damage caused by the intrinsic instability of DNA and by oxidative metabolism (Robertson et al., 2009). The amount of such damage in normal cells is substantial with as many as 50,000 BER lesions formed daily. These spontaneously generated DNA adducts, including oxidized and alkylated bases, abasic sites, and single-strand breaks (SSBs), are identical to those generated by therapeutic ionizing radiation and many clinically utilized alkylating agents.

**FIGURE 1:**
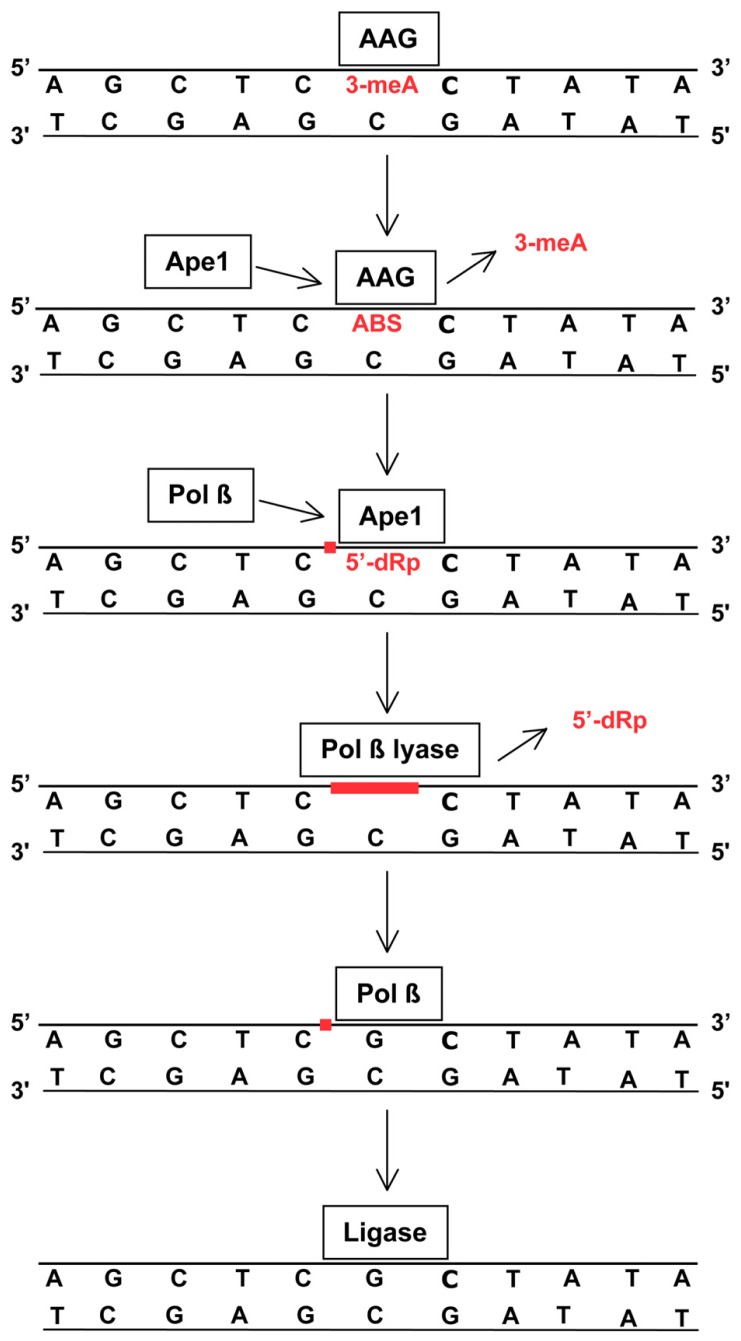
**Removal of 3-meA by base excision repair (BER)**. TMZ-induced 3-meA in DNA is recognized by AGG that excises the methylated base by cleaving the glycosylic linkage between the adducted base and deoxyribose. AAG remains bound to the resulting abasic site (ABS) until displaced by Ape1. The apurinic endonuclease activity of Ape1 incises the DNA phosphodiester backbone immediately 5′ to the abasic site, producing a single-strand break (red square) containing a 5′-deoxyribose phosphate (5′-dRP) terminus. Ape1 is replaced by DNA polymerase β(Pol β) that removes the 5′-dRP via an intrinsic lyase activity to produce a single-nucleotide gap (red line). Using the opposite DNA strand as a template, Pol βthen inserts a complementary nucleotide leaving a single-strand break (red square) that is subsequently sealed by DNA ligase. This process is closely coordinated to insure that the potentially lethal abasic sites, and single strand breaks and gaps do not persist. XRCC1 and PARP (not shown) facilitate repair by coordinating protein binding at damage sites. BER is also essential for repair of abasic sites that arise from spontaneous or methylation-enhanced base loss and single-strand breaks produced by ionizing radiation. Some radiation-induced strand breaks contain fragmented or 5′-oxidized deoxyribose moieties that cannot be excised by the lyase activity of Pol β. In this circumstance an alternative BER pathway employs FEN1 endonuclease to excise a two to eight nucleotide long single-strand containing the damage, and the resulting gap is filled by DNA polymerase δ or ε and sealed by DNA ligase (Robertson et al., 2009).

The BER of 3-meA and 7-meG is initiated by adduct recognition by AAG (also methylpurine-DNA glycosylase or alkylpurine-DNA-*N*-glycosylase) followed by cleavage of the glycosylic linkage between the damaged base and deoxyribose, producing an abasic site in DNA (**Figure 1**). AAG remains bound to the abasic site and recruits Ape1, the major human Ap endo (Abbotts and Madhusudan, 2010), which cleaves the DNA phosphodiester backbone to form a SSB with 3′-OH and 5′ deoxyribose phosphate (dRP) termini. Ape1 is then replaced by DNA polymerase β, a repair polymerase possessing a 5′ lyase activity that excises the 5′ dRP to yield a single nucleotide gap. The gap is filled by DNA polymerase β, leaving a strand break that is immediately sealed by DNA ligase III. The tightly coordinated mechanism of BER, necessary prevent accumulation of potentially lethal repair intermediates (i.e., abasic sites, 5′ dRPs, SSBs), is mediated by the protein XRCC1 which serves as a scaffold to foster the sequential action of each repair enzyme. In addition, poly(ADP-ribose) polymerase (PARP) facilitates repair by binding to SSBs and synthesizing long poly(ADP-ribose) chains that recruit XRCC1, DNA polymerase β, and DNA ligase to the site of repair. The coordinated action of BER components is essential for methylator resistance, as evidenced by the increased cytotoxicity that accompanies unbalanced repair produced either by suppressed or enhanced expression of key elements (Fu et al., 2012).

Short patch BER is not the sole mechanism in human cells that promotes methylator resistance by removing N-methylated bases from DNA. 1-meA and 3-meC are demethylated *in situ* by the DNA dioxygenases ABH2 and ABH3 (Sedgwick et al., 2007). Other mechanisms promote tolerance of unrepaired lesions, e.g., translesion synthesis by Y family DNA polymerases (Plosky et al., 2008; Monti et al., 2010), replication restart at stalled forks (Blank et al., 2004), and rejoining of DSBs arising at sites of blocked DNA replication by homologous recombination and non-homologous end joining (Nikolova et al., 2010; Quiros et al., 2011). Full characterization of the contribution of these additional mechanisms to TMZ resistance in human gliomas awaits further study.

## AAG PROMOTES RESISTANCE TO TMZ

Bacteria, yeast, and mammalian cells unable to excise 3-meA are hypersensitive to laboratory and clinically utilized methylating agents (Fronza and Gold, 2004; Wyatt and Pittman, 2006). Evidence that repair of 3-meA mediates alkylator resistance in human cancer cells has come from experiments in which AAG expression was suppressed and from studies using unique, sequence-specific alkylators that produce 3-meA as their sole cytotoxic lesion (Fronza and Gold, 2004). Below, we discuss the evidence that repair of 3-meA by AAG contributes to TMZ resistance in human GBM cells and tumors.

## AAG SUBSTRATE SPECIFICITY AND PHYSIOLOGICAL ROLE 

Alkyladenine-DNA glycosylase is one of 11 human DNA glycosylases characterized to date, and appears to be the primary activity that excises 3-meA and 7-meG from DNA (Fu et al., 2012). Unlike most DNA glycosylases, AAG has broad substrate specificity that includes oxidized and alkylated bases (Wyatt and Pittman, 2006). Hypoxanthine, a mutagenic deamination product of adenine, is the preferred substrate and is excised at least 1,000-fold more efficiently than alkyl adducts (O’Brien and Ellenberger, 2004; Lee et al., 2009). Of the *N*-methylpurines, 3-meA is excised at a greater rate than 7-meG, and there is no evidence suggesting that AAG excises other methylated purines, or methylated pyrimidines *in vivo*. The catalytic activity of AAG against *N*-methylpurines is very modest in that, compared to spontaneous depurination, it reduces the half-lives for 3-meA and 7-meG in DNA only about a 1,000-fold (i.e., from hours to minutes), far less than the >10^20^ rate enhancements yielded by most enzymes (O’Brien and Ellenberger, 2004). Excision of 7-meG, an innocuous adduct, by AAG to yield a cytotoxic abasic site could be a consequence of the broad substrate specificity of the enzyme; however, such excision may confer a selective advantage by preventing spontaneous depurination that would yield an unprotected abasic site. Of note, other AAG substrates include the DNA blocking adducts bases 1,N^6^-ethanoadenine and 1,N^6^-ethenoadenine produced by 1,3-bis(2-chloroethyl)-1-nitrosourea (BCNU) and cyclophosphamide, respectively, two agents used to treat recurrent GBMs (e.g., Chamberlain and Tsao-Wei, 2004; Stupp et al., 2007).

Mice homozygous null for *Aag* are viable, develop normally and do not display enhanced rates of spontaneous carcinogenesis. However, *Aag*^–/–^ animals and primary embryonic fibroblasts do show increased sensitivity to methylating agents (Engelward et al., 1998). In accord, suppressing AAG expression with siRNA produced hypersensitivity to TMZ and other alkylators in HeLa and ovarian carcinoma cells (Paik et al., 2005). Yet, the importance of AAG in countering methylator genotoxicity in normal cells is not unambiguous, as evidenced by results showing that loss of activity is not necessarily accompanied by greater methylator sensitivity in some cell types (Wyatt and Pittman, 2006). For example, *Aag*^–/–^ mouse myeloid bone marrow cells are more resistant to methylator-induced killing than wild-type cells, suggesting that excision of methyl adducts by AAG might promote killing. Notably, over-expression of AAG in rodent and human cancer cells increases sensitivity to methylators, including TMZ, that has been attributed to an imbalance in BER resulting in accumulation of cytotoxic abasic sites (e.g., Rinne et al., 2005; Tang et al., 2011). As discussed below, greater TMZ resistance accompanies high levels of AAG expression in human GBM tissue, indicating that unbalanced BER does not commonly accompany glial tumorigenesis.

## AAG PROMOTES TMZ RESISTANCE IN HUMAN GBM AND GLIOMA CELLS 

Early work from our laboratory strongly indicated that DNA adducts in addition to O^6^-meG cause TMZ cytotoxicity in human GBM cell lines, and that MGMT is not the only, or even the principal, agent of TMZ resistance (Bobola et al., 1996). We addressed this hypothesis directly by examining the sensitivity of a panel of 10 human glioma cell lines to methyl-lexitropsin (Me-Lex), a novel, sequence-specific alkylating agent that produces 3-meA as the predominant (i.e., >90%) base adduct (Fronza and Gold, 2004). Suppressing AAG activity with antisense oligonucleotides (ASO) in MGMT-expressing, MGMT-deficient and MGMT- and mismatch repair-deficient GBM lines increased Me-Lex sensitivity assayed by survival of colony-forming ability (Bobola et al., 2007). Importantly, greater cell killing was accompanied by reduced content of abasic sites, the product of AAG-mediated excision of 3-meA. These finding provide strong evidence that unrepaired 3-meA is a potentially lethal lesion in human glioma cells and suggest that 3-meA plays a role in TMZ-induced cell killing.

Further evidence that TMZ-induced 3-meA contributes to cell killing was described in recent work examining the TMZ sensitivity of A172 cells, a human GBM cell line that has no detectable AAG or MGMT protein by Western blotting (Agnihotri et al., 2012). In these experiments variant lines were constructed that either expressed AAG alone, MGMT alone, or both repair activities. The variant line expressing AAG alone displayed elevated resistance to TMZ that was accompanied by decreased content of 7-meG, relative to the repair-deficient parental line. Co-expression of AAG and MGMT resulted in yet greater TMZ resistance, consistent with independent contributions of AAG and MGMT to resistance. In complementary experiments, Agnihotri et al. (2012) showed that shRNA-mediated suppression of endogenous AAG increased sensitivity to TMZ in the GBM cancer stem cell line GBM6, and to methyl methanesulfonate (MMS), a methylator that produces very little O^6^-meG, in the GBM line T98G. More recently, our laboratory found that ASO-mediated suppression of AAG activity increased TMZ killing in MGMT-expressing, MGMT-deficient and MGMT- and mismatch repair-deficient GBM lines (Bobola et al., in preparation), providing additional evidence that TMZ-induced 3-meA promotes cytotoxicity in GBM cells. Elevated content of γ-H2AX, a surrogate marker for DSBs (Bonner et al., 2008), accompanied greater sensitivity to TMZ in ASO-treated cells, suggesting that unrepaired 3-meA is a precursor of lethal DSBs.

A role for AAG in TMZ resistance was further supported by examination of a panel of 19 xenografts established by intra-cranial implantation of human GBM tissue in nude mice (Agnihotri et al., 2012). AAG expression was undetectable by immunohistochemistry (IHC) in 11 of 19 (58%) xenografts, and absence of AAG was accompanied by significantly longer survival following treatment with TMZ. Comparable results were observed for survival of nude mice bearing intra-cranial xenografts derived from the A172 variant lines following treatment with TMZ, yielding strong evidence that 3-meA and O^6^-meG contribute independently to TMZ cytotoxicity. Interestingly, TMZ sensitivity was the same in variants expressing AAG alone or MGMT alone, suggesting that unrepaired 3-meA and O^6^-meG were equally cytotoxic.

A growing body of evidence indicates that TMZ is a radiosensitizing agent and that this property is partly responsible for the clinical efficacy of concomitant treatment with TMZ and radiation (Silber et al., 2012). Radiosensitization is most evident in MGMT-deficient GBM cells leading to the conclusion that failure to repair O^6^-meG promotes radiation sensitivity. In addition, we have reported that ASO-mediated suppression of AAG activity enhanced radiation killing in MGMT-expressing and MGMT- and mismatch repair-deficient GBM cells treated with minimally cytotoxic doses of TMZ (Bobola et al., 2010), indicating that 3-meA also promotes radiosensitization. Comparable increases in radiosensitivity were obtained when cells were exposed to Me-Lex, a methylator that produces almost exclusively 3-meA (Bobola et al., in preparation). In accord with these findings is the recent report that nude mice bearing AAG-deficient A172 xenografts survived significantly longer following concurrent treatment with TMZ and radiation compared to xenografts of AAG-expressing A172 variants (Agnihotri et al., 2012). These results strongly indicate that 3-meA as well as O^6^-meG contribute to TMZ-induced radiosensitization.

## AAG IN GLIOMA TISSUE AND ASSOCIATION WITH TREATMENT RESPONSE

A number of studies have shown that gliomagenesis is accompanied by elevation of AAG expression as demonstrated by greater AAG mRNA content in gliomas relative to adjacent brain (Kim et al., 2003; Tang et al., 2011; Liu et al., 2012). IHC analysis of GBM and other gliomas revealed predominantly nuclear expression of AAG protein that displayed considerable inter-tumoral heterogeneity, with 20–30% of specimens having no detectable protein (Agnihotri et al., 2012; Liu et al., 2012). The analysis by Agnihotri et al. (2012) revealed that absence of detectable protein is associated with extensive methylation of promoter CpG islands, suggesting that AAG expression is epigenetically regulated in GBMs. Our recent analysis of AAG activity in 80 GBMs revealed a greater than a 820-fold range in activity (Bobola et al., in preparation). Only four tumors (~6%) lacked detectable activity, likely reflecting the greater sensitivity of biochemical assay compared to IHC for detection of AAG.

Agnihotri et al. (2012) found that AAG expression, assessed by IHC, is inversely associated with overall survival in GBMs. In a sample of 37 GBMs, patients with immunonegative tumors had significantly longer overall survival following concurrent therapy with TMZ and radiation than patients with AAG-expressing tumors. Examination of AAG protein expression in another set of 27 GBMs displaying MGMT promoter methylation, a marker of low or absent MGMT expression, revealed that tumors with overall survival >1 year were significantly more likely to lack detectable AAG compared to tumors with shorter survival. These data suggest that AAG promotes resistance to TMZ-RT independently of MGMT. In accord with this conclusion, analysis of a larger set of tumor samples from the EORTC-NCIC trial (Stupp et al., 2009) revealed that undetectable AAG correlated with significantly longer overall survival (*P* = 0.04) in GBMs treated with concurrent TMZ-RT, regardless of MGMT promoter methylation status. That this relationship reflected repair of TMZ-induced DNA damage was evidenced by lack of an association between AAG expression and survival in GBMs treated with radiation only.

Our laboratory has recently completed a preliminary examination of the association of AAG biochemical activity with progression-free survival (PFS) following alkylator treatment in 60 *de novo* GBMs that differed in activity by 820-fold. Forty-three tumors were treated with radiation followed by alkylator-based chemotherapy, including 23 that received TMZ. The remaining 17 tumors were treated with concurrent TMZ-RT. A dichotomous Cox proportional hazards regression model with median activity as the cut point revealed a strong inverse trend between activity and PFS with the risk of progression changing by a factor of 1.64 for each unit of AAG activity (*P* = 0.082). Including MGMT activity as a covariate yielded a significant association with a hazard ratio (HR) = 1.96 (*P* = 0.033), suggesting that AAG and MGMT independently promote alkylator resistance. Comparable trends were observed when analysis was restricted to the 43 tumors treated with TMZ (HR = 1.90; *P* ≤ 0.10) and the 17 treated with concurrent TMZ-RT (HR = 3.42; *P* ≤ 0.15). The inverse association was also observed when AAG activity was entered as a continuous variable in a univariate Cox model (HR = 1.002; *P* ≤ 0.047); addition of MGMT activity as a covariate strengthened the association (HR = 1.003; *P* ≤ 0.015), suggesting that AAG and MGMT contribute independently to resistance. In the bivariate model, the difference in risk of progression between the GBMs with the lowest and highest AAG activity was approximately 12-fold (i.e., 1.003^820^).

## Ape1 PROMOTES RESISTANCE TO TMZ

Bacterial and yeast mutants lacking repair activities analogous to human Ape1 and cells from *Ape1* heterozygous mice are hypersensitive to laboratory and clinically utilized methylating agents (Evans et al., 2000; Abbotts and Madhusudan, 2010). Ape1 contributes to methylator resistance in a variety of human tumor cells as demonstrated by experiments showing that suppressing Ape1-mediated Ap endo activity increased drug sensitivity while increasing activity promoted resistance (Evans et al., 2000; Abbotts and Madhusudan, 2010). Below, we discuss the evidence that repair of abasic sites by Ape1 contributes to TMZ resistance in human GBM cells and tumors.

## Ape1 ENZYMATIC ACTIVITIES AND FUNCTION

Ape1 (also Ape1/Ref-1) is a multifunctional protein that is ubiquitously expressed in human cells. Ape1 fosters cell survival by participating in repair of endogenously and exogenously induced cytotoxic DNA lesions and by activating transcription factors that promote resistance to stress (Abbotts and Madhusudan, 2010). Ape1 also participates in the regulation of calcium-dependent gene expression and has roles in processing rRNA and mRNA. At least some of these Ape1-mediated functions are necessary for survival, as evidenced by the early embryonic lethality of *APE1* knockout mice (Izumi et al., 2005). The multi-functionality of Ape1 is reflected in its unusual abundance (>10^5^ copies per cell) and its distribution in cytoplasm and mitochondria as well as the nucleus.

Ape1 possesses a strong Ap endo activity that accounts for more than 95% of the abasic site incision activity observed in human cells (reviewed in Tell et al., 2009; Abbotts and Madhusudan, 2010). In addition to Ap endo activity, Ape1 also has 3′-phosphodiesterase, 3′-phosphatase, and 3′-exonuclease activities that are critical for repair of abasic sites and SSBs containing fragmented 3′-deoxyribose or 3′-phosphate induced by the reaction of oxidative free radicals at DNA bases and deoxyribose, accounting for a role for Ape1 in resistance to ionizing radiation. All of these activities are catalyzed by a common active site located in the C-terminus of the protein. The N-terminus of Ape1 is the redox protein Ref-1 that participates in response to DNA damage-induced stress, cell cycle control and apoptosis by maintaining critical transcription factors in an active, reduced state. Ref-1 has also been implicated in regulating the transactivation and proapoptotic functions of p53. Notably, exogenous oxidative stress, including hypoxia and ionizing radiation, transiently elevate Ape1 protein content and Ap endo activity and increase alkylating agent resistance in human tumor cell lines (e.g., Silber et al., 2002). These properties indicate that Ape1 plays a critical role in GBM resistance to adjuvant radiation and alkylating agents, a conclusion supported by the evidence presented below.

## Ape1 PROMOTES RESISTANCE TO TMZ IN GBM CELLS

In early experiments, Ono et al. (1994) found that suppressing Ap endo activity in rat glioma cells by using antisense expression constructs was accompanied by greater sensitivity to the methylating agent MMS and to the oxidizing agent hydrogen peroxide. In subsequent work, the same group reported that sensitivity to MMS and hydrogen peroxide was inversely correlated with Ap endo activity in a panel of human glioma-derived cell lines (Ono et al., 1995). These experiments, however, provided no information as to which Ape1 function promoted resistance. To address this question and to further investigate the role of Ape1 in alkylator resistance, we used anti-Ape1 ASO to show that suppressing Ap endo activity in the MGMT-deficient human GBM line SNB19 increased sensitivity to TMZ (Silber et al., 2002). TMZ hypersensitivity was accompanied by elevated abasic site content, indicating that failure to excise abasic sites contributed, at least in part, to the potentiation of cell killing. Conversely, elevating Ap endo activity by exposing cells to minimally cytotoxic oxidative stress increased TMZ resistance and reduced abasic site content. Comparable effects on survival were observed for cells treated with the chloroethylating agent 1,3-bis(2-chloroethyl)-1- nitrosourea (BCNU, carmustine). In subsequent experiments, we showed that suppression of Ap endo activity with anti-Ape1 ASO increased killing by the sequence-specific methylator Me-Lex in MGMT-proficient, MGMT-deficient, and MGMT- and mismatch repair-deficient GBM lines (Bobola et al., 2007). Increased cytotoxicity was accompanied by elevated abasic site content, indicating that lack of Ape1-mediated repair of abasic sites arising from excision of 3-meA promoted cell killing. Together, these two studies provide evidence that failure to excise abasic sites derived from TMZ-induced *N*-methylpurines contributes to GBM cell killing regardless of the ability to repair or tolerate O^6^-meG. A potential role for Ape1 in promoting resistance to adjuvant therapy in primary brain tumors in addition to GBMs is supported by our findings that suppressing Ape1 expression and Ap endo activity increases the sensitivity of pediatric ependymoma cells to radiation (Bobola et al., 2011) and medulloblastoma cells to TMZ and BCNU (Bobola et al., 2005). In the latter case, TMZ sensitivity was also increased in MGMT- and mismatch-repair deficient medulloblastoma cells that are insensitive to the lethality of unrepaired O^6^-meG.

## Ape1 IN GLIOMA TISSUE AND ASSOCIATION WITH TREATMENT RESPONSE

Ape1 expression has been the subject of intensive investigation in a number of human cancers, including primary brain tumors (Bobola et al., 2004, 2005, 2011). In an initial study of human adult gliomas (Bobola et al., 2001), our laboratory assayed Ap endo activity in 84 tumors to establish correlates with tumor characteristics, and in histologically normal brain adjacent to 58 of the tumors to characterize changes in activity accompanying neurocarcinogenesis. Activity in all gliomas ranged *ca*. 550-fold and was, on average, 3.5-fold greater in anaplastic gliomas and GBMs than in low-grade tumors, suggesting that proliferation may be a determinant of activity. In accord, Ap endo activity was positively correlated with the fraction of S-phase cells. In the 58 cases of tumor paired with adjacent normal brain, mean activity was more than sevenfold higher in tumor than in normal tissue. Increased tumor activity was observed in 93% of tumor/normal pairs, indicating that elevation of Ap endo activity is characteristic of human gliomagenesis. The elevation was large in most pairs, being 13-fold on average and ≥10-fold in 43% of cases. A concomitant increase in Ape1 protein was observed by Western blotting in the subset of tumor/normal pairs examined. These findings suggest that the increase in Ap endo activity that accompanies gliomagenesis could enhance resistance to adjuvant therapy for GBM and other gliomas.

Numerous reports have described an inverse association between immunopositivity for Ape1 and clinical course in a variety of human tumors (Evans et al., 2000; Abbotts and Madhusudan, 2010). To extend these studies to human gliomas and to evaluate Ap endo activity as a marker of treatment response, we examined the association of Ap endo activity with PFS following sequential treatment with radiation and alkylating agents in 30 anaplastic gliomas and 34 GBMs (Bobola et al., 2004). Cox regression analysis with Ap endo activity entered as a continuous variable revealed an inverse relationship with a HR for progression following alkylator therapy in the anaplastic gliomas increasing by a factor of 1.061 for every 0.01 increase in activity (*P* = 0.013). In contrast, we observed no association between activity and PFS in the GBMs, a result we attributed, in part, to the narrow range of PFS displayed by the tumors.

More recently, we have analyzed the association between Ap endo activity and PFS following treatment with alkylating agents in 80 *de novo* GBMs that differed in Ap endo activity by ~225-fold (Bobola and Silber, in preparation). Sixty-four tumors were treated with radiation followed by alkylating agent-based chemotherapy and 16 were treated with concurrent TMZ-RT. A dichotomous Cox regression model revealed a twofold greater risk of progression (HR = 2.07; *P* ≤ 0.003) for tumors with greater than median Ap endo activity. Analyzing Ap endo as a continuous variable revealed that the risk of progression increased by a factor of 1.050 for every 0.01 unit increase in activity (*P* ≤ 0.022). In this group of GBMs, the difference in risk of progression between the tumor with the highest and lowest Ap endo activity was 3.3-fold. Analyses of 65 anaplastic gliomas that differed in Ap endo activity by 760-fold revealed a 2.1-fold greater risk for progression associated with activities greater than the median (*P* ≤ 0.022), and a 1.035 increase in risk for each 0.01 unit increase in activity (*P* ≤ 0.005) indicative of an 8.7-fold difference in risk between the tumors with the lowest and highest activities. These findings strongly indicate that Ap endo activity promotes resistance to alkylator agent therapy in GBMs and anaplastic gliomas. They also suggest that Ap endo activity may have utility as a marker of treatment response and is a potential target for anti-resistance therapies.

## CIRCUMVENTING REPAIR OF ABASIC SITES TO REDUCE TMZ RESISTANCE

The association of DNA repair with clinical response to therapeutic DNA damaging agents has provided strong impetus to develop inhibitors of repair to improve treatment outcome. Characterization of inhibitors of MGMT (e.g., O^6^-benzylguanine, lomeguatrib) and of PARP1 (e.g., olaparib; ABT-888) to circumvent resistance to TMZ are paradigms for this strategy (Mrugala and Chamberlain, 2008; Leonetti et al., 2012).

The role of Ape1 in promoting resistance to adjuvant therapy in GBM and other gliomas has stimulated interest in developing small molecule inhibitors targeting Ap endo activity (Wilson and Simeonov, 2010). Lucanthone, a DNA intercalator used to treat schistosomiasis (Bases and Mendez, 1997), was the first potential inhibitor identified. Lucanthone has been reported to inhibit incision at abasic sites by Ape1 *in vitro* and increase abasic site content in HeLa cells (Mendez et al., 2002), and to increase TMZ sensitivity in human breast cancer cells (Luo and Kelley, 2004) and human GBM cells (Silber and Bobola, unpublished observations). The mechanism of action was initially believed to involve drug intercalation that obscures abasic sites, but more recent evidence indicates that lucanthone acts, at least in part, by binding to the active site of Ape1 (Naidu et al., 2011). Lucanthone has also been shown to sensitize brain metastases to radiation (Del Rowe et al., 1999). On the basis of this sensitization, together with demonstrated safety and ability to cross the blood brain barrier (Del Rowe et al., 1999), lucanthone is currently in phase II trial (NCT01587144) to evaluate safety and efficacy in GBMs treated with concurrent TMZ-RT.

Ongoing investigation using high-throughput screening coupled with molecular modeling of active site binding has identified a large number of potential small molecule inhibitors of Ape1-catalyzed Ap endo activity (Bapat et al., 2010; Wilson and Simeonov, 2010; Mohammed et al., 2011; Rai et al., 2012). While all of these compounds inhibit Ap endo activity *in vitro*, only a limited number have been shown to sensitize cells to TMZ. Madhusudan et al. (2005) identified inhibitors from a library of over 2.5 million compounds (e.g., 7-nitro-indole-2-carboxylic acid) that sensitized fibrosarcoma and GBM cells to MMS and TMZ (Madhusudan et al., 2005; Mohammed et al., 2011). Bapat et al. (2010) identified four potential inhibitors, including one (AR03) that elicited hypersensitivity to MMS and TMZ in the malignant glioma line SF767. Both groups reported that methylator hypersensitivity was accompanied by elevated abasic site content, affording evidence of inhibition of Ap endo activity *in vivo*. More recently, a series of Ape1 Ap endo inhibitors based on 2-methyl-4-amino-6,7-dioxolo-quinoline was shown to increase sensitivity to the methylator Me-Lex and to concomitantly increase abasic site content in the human GBM line T98G (Srinivasan et al., 2012). Also, Rai et al. (2012) have described synthesis and characterization of the inhibitor, *N*-(3-(benzo[d]thiazol-2-yl)-6-isopropyl-4,5,6,7 tetrahydrothieno[2,3-c)pyridin-2yl)acetamide that sensitized HeLa cells to MMS and TMZ. Of note, this lipophilic compound readily crossed the blood-brain barrier of mice, satisfying one requirement for efficacy in treating human gliomas.

To our knowledge there are no small molecule inhibitors of AAG in development. However, the advent of site-specific methylators that produce 3-meA at ~ninefold higher yields than TMZ (Fronza and Gold, 2004) suggest that it may be possible to induce numbers of adducts that are sufficient to overwhelm tumor cell repair capacity.

## CONCLUSIONS AND FUTURE CONSIDERATIONS

The demonstration that *MGMT* promoter methylation status is associated with GBM response to TMZ-based therapies highlights the potential importance of DNA repair in determining clinical course (Silber et al., 2012). However, methylation status does not accurately predict treatment response in the majority of GBM, suggesting that MGMT is not the sole, or even the predominant, determinant of therapeutic response. As set out in this review, there is now increasing evidence that AAG and Ape1 also promote resistance in GBMs treated with TMZ and other alkylators, supporting the multifactorial nature of DNA repair-mediated treatment failure in GBMs. The clinical relevance of repair of 3-meA and abasic sites is illustrated in **Table 2** which documents our finding that the association of tumor AAG and Ap endo activities with alkylating agent response is comparable to that of MGMT. The goal now is to translate this knowledge into more effective treatments for GBM.

**Table 2:** Association of AAG, Ape1, and MGMT activity with GBM resistance to radiation and alkylating agents: one laboratory’s experience^a^.

Repair activity^b^	*N*^c^	HR	*B* =
AAG	60	1.64	0.082
Ape1	80	2.07	0.003
MGMT	87	1.90	0.006

a Risk for progression following radiation and alkylator therapy was determined by Cox proportional hazards regression in dichotomous models with median activity as the cut point. See text for details

b Activities in GBMs were measured by biochemical assay.

c Tumors assayed for AAG and Ape1 were from the sample of 87 assayed for MGMT.

The emerging data reviewed here suggest that that AAG and Ape1 may have utility as markers of clinical response to TMZ-based adjuvant therapy. Inclusion of AAG and/or Ape1 together with MGMT expression in multivariate models may allow more accurate prediction of clinical response and further the goal of individualizing treatment for GBM, an expectation supported by the stratification of survival based on AAG immunopositivity in both *MGMT* promoter methylated and unmethylated GBMs (Agnihotri et al., 2012). Realizing this goal will require development of clinically tractable assays for these proteins. Assay of AAG and Ape1-mediated Ap endo activity is not currently practical for routine clinical laboratory use. IHC and surrogate measures of gene expression, such as promoter methylation status, have the advantage of using fixed tissue as starting material. In the case of AAG, both of these approaches may be efficacious, as evidenced by the stratification of survival based on AAG immunopositivity mentioned above, and initial evidence that AAG expression is inversely associated with methylation of promoter CpG islands (Agnihotri et al., 2012). Ape1 expression is particularly attractive as a marker of GBM clinical outcome because of its multiple DNA repair and non-repair functions that promote resistance to radiation as well as alkylating agents (Abbotts and Madhusudan, 2010). Numerous studies have associated Ape1 immunopositivity with clinical response in a variety of human cancers, suggesting that this measure may prove useful in GBM and other adult gliomas.

AAG and Ape1 are also attractive targets for anti-resistance therapies to enhance the effectiveness of TMZ and other alkylators. As discussed, suppressing AAG or Ape1 expression is accompanied by greater alkylator sensitivity in human GBM cells regardless of their ability to repair or tolerate cytotoxic O^6^-meG. The development of small molecule inhibitors of the Ap endo activity of Ape1 and of sequence-specific alkylators that produce 3-meA as the sole cytotoxic lesion are active areas of investigation. The promising recent results suggesting the potential of these strategies to improve clinical outcome must be tempered by the difficulty in translating preclinical findings with GBM cells into effective human therapies. Clinical utilization requires circumventing numerous pharmacological limitations, including stability, solubility, excretion, and ability to penetrate physiological barriers. In addition, systemic administration of inhibitor molecules increases the risk of producing unacceptable off-target toxicity that leads to efficacy-compromising alkylator dose reductions. Circumventing these problems will require pharmacologically compatible delivery vehicles that sequester inhibitor during transit, penetrate the blood-brain barrier and specifically target tumor cells. Prototypes of such agents have been described, but this area of research is still in its infancy.

## Conflict of Interest Statement

The authors declare that the research was conducted in the absence of any commercial or financial relationships that could be construed as a potential conflict of interest.
